# First-principles investigation of a type-II BP/Sc_2_CF_2_ van der Waals heterostructure for photovoltaic solar cells

**DOI:** 10.1039/d3na00082f

**Published:** 2023-03-28

**Authors:** Nguyen Dang Khang, Cuong Q. Nguyen, Le M. Duc, Chuong V. Nguyen

**Affiliations:** a Faculty of Electrical Engineering, Hanoi University of Industry Hanoi 100000 Vietnam khangnd@haui.edu.vn; b Institute of Research and Development, Duy Tan University Da Nang 550000 Vietnam nguyenquangcuong3@duytan.edu.vn; c Faculty of Natural Sciences, Duy Tan University Da Nang 550000 Vietnam; d Department of Materials Science and Engineering, Le Quy Don Technical University Hanoi Vietnam chuong.vnguyen@lqdtu.edu.vn

## Abstract

Constructing heterostructures has proven to be an effective strategy to manipulate the electronic properties and enlarge the application possibilities of two-dimensional (2D) materials. In this work, we perform first-principles calculations to generate the heterostructure between boron phosphide (BP) and Sc_2_CF_2_ materials. The electronic characteristics and band alignment of the combined BP/Sc_2_CF_2_ heterostructure, as well as the effects of an applied electric field and interlayer coupling, are examined. Our results predict that the BP/Sc_2_CF_2_ heterostructure is energetically, thermally and dynamically stable. All considered stacking patterns of the BP/Sc_2_CF_2_ heterostructure possess semiconducting behavior. Furthermore, the formation of the BP/Sc_2_CF_2_ heterostructure gives rise to the generation of type-II band alignment, which causes photogenerated electrons and holes to move in opposite ways. Therefore, the type-II BP/Sc_2_CF_2_ heterostructure could be a promising candidate for photovoltaic solar cells. More interestingly, the electronic properties and band alignment in the BP/Sc_2_CF_2_ heterostructure can be tuned by applying an electric field and modifying the interlayer coupling. Applying an electric field not only causes modulation of the band gap, but also leads to the transition from a semiconductor to a gapless semiconductor and from type-II to type-I band alignment of the BP/Sc_2_CF_2_ heterostructure. In addition, changing the interlayer coupling gives rise to modulation of the band gap of the BP/Sc_2_CF_2_ heterostructure. Our findings suggest that the BP/Sc_2_CF_2_ heterostructure is a promising candidate for photovoltaic solar cells.

## Abstract 2

We have constructed a BP/Sc_2_CF_2_ heterostructure and explored its electronic properties and band alignment, as well as the effects of applying an electric field and modifying the interlayer coupling, using first-principles prediction.

## Graphical abstract

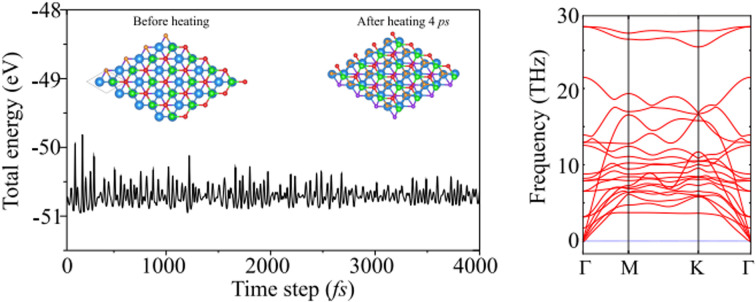

## 1 Introduction

Two-dimensional (2D) materials have gained considerable interest from the scientific community owing to their superior properties and promising applications for future high-efficiency devices.^1,2^ Starting from the discovery of graphene in 2004,^3^ many 2D materials have been predicted and explored, including hexagonal boron nitride (h-BN),^4^ transition-metal dichalcogenides (TMDCs)^5,6^ and phosphorene.^7,8^ Although 2D materials possess many intriguing physical and chemical properties, they have some drawbacks that may hinder their applications in some nanodevices. For instance, the lack of a band gap in graphene hinders its high-speed applications, including field-effect transistors (FET).^9^ The structural instability of phosphorene under ambient conditions hinders its use in practical applications.^10^ Therefore, the search for novel 2D materials with desired properties that merit the requirements of practical applications is still challenging.

Recently, a new type of 2D material, called MXenes, has been developed and synthesized experimentally by selectively etching “A” layers from transition-metal carbide MAX phases.^11^ Nowadays, many different types of MXenes have been predicted and investigated.^12–16^ Most 2D MXenes possess metallic characteristics.^17^ Interestingly, surface functionalization can be used to transform some MXene materials from a metal to a semiconductor, including Sc_2_CX_2_ (X = F, O and OH).

Furthermore, boron phosphide (BP), a group III–V material with a planar honeycomb structure has received much interest from the research community owing to its unique characteristics, including high carrier mobility^18^ and controllable electronic properties.^19^ Monolayer BP presents semiconducting behavior with a band gap ranging from 1 to 2 eV, depending on the calculated methods.^20^ Moreover, the BP monolayer is proven to be thermally and dynamically stable.^21^ Interestingly, high-quality BP films have been successfully synthesized using the chemical vapor deposition (CVD) approach.^22^ Following developments in science and technology, BP monolayers have been synthesized in recent experiments.

Currently, several strategies have also been developed to manipulate the physical properties of 2D materials, such as doping,^23,24^ applying strain^25,26^ and constructing heterostructures.^27,28^ Among those, it is interesting that constructing heterostructures has proved to be an effective strategy to manipulate the electronic properties and increase the application possibilities of 2D materials. The heterostructures can be constructed and predicted both theoretically and experimentally by stacking a 2D material on top of another. Experimentally, heterostructures can be synthesized by several common methods, including both top–down and bottom–up methods,^29–31^ whereas the heterostructures can be generated theoretically by first-principles methods.^32–34^ Compared to the parent 2D materials, their heterostructures exhibit many more intriguing properties that are advantageous for the design of high-efficiency nanodevices. For instance, a heterostructure comprising two different 2D materials generates different band alignments, including type-I (straddling gap), type-II (staggered gap) and type-III (broken gap). The generation of different types of band alignments in heterostructures makes them suitable for different varieties of applications, including light-emitting diodes^35^ for type-I heterostructures, optoelectronics and photocatalysis for type-II heterostructures^36^ and field-effect transistors for type-III heterostructures.^37^

Interestingly, the combination between a BP monolayer and different 2D materials may give rise to the generation of type-I^38,39^ and type-II^39–41^ heterostructures. Similarly, the combination between a Sc_2_CF_2_ monolayer and other 2D materials can also result in the formation of heterostructures with different types of band alignment, such as type-I, type-II or type-III heterostructures.^42,43^ However, to date, the combination between BP and Sc_2_CF_2_ materials has not yet been constructed and investigated thoroughly. Therefore, in this work, we construct heterostructures between BP and Sc_2_CF_2_ monolayers using first-principles calculations and investigate the electronic properties and band alignment of BP/Sc_2_CF_2_ heterostructures.

## 2 Computational details

The first-principles calculations are performed in the Quantum Espresso (PWscf) simulation package^44^ using the projected augmented wave (PAW) method.^45^ The exchange-correlation energy is described by adopting the Perdew–Burke–Ernzerhof (PBE) functional within the generalized gradient approximation (GGA).^46,47^ For the self-consistent calculations, the energy and forces are converged to be 10^−6^ eV and 0.01 eV Å^−1^, respectively. A 9 × 9 × 1 *k*-point mesh is applied for optimization and electronic structure calculations. The cut-off energy is set to be 510 eV. The semi-empirical dispersion correction proposed by Grimme, called the DFT-D3 method, is adopted for modifying the long-range vdW interactions in layered heterostructures.^48^ A vacuum thickness of 25 Å is applied to prevent the interactions between adjacent layers caused by periodicity. The HSE06 (Heyd–Scuseria–Ernzerhof) hybrid functional^49^ is employed to obtain more accurate band gaps of the materials.

## 3 Results and discussion

**Fig. 1:**
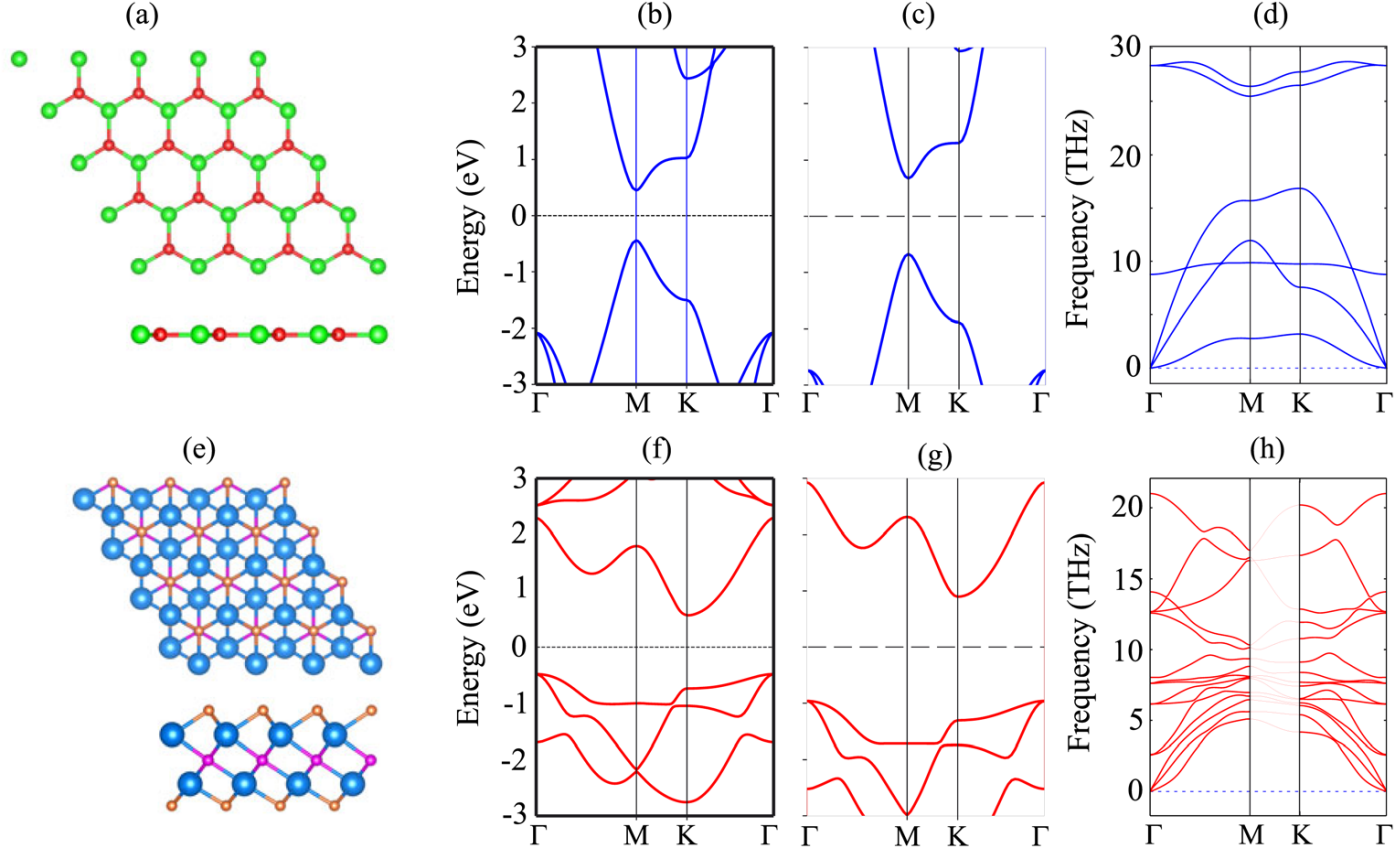
(a and e) Atomic structures, (b and f) PBE and (c and g) HSE band structures and (d and h) phonon spectra of (a–d) BP and (e–h) Sc_2_CF_2_ monolayers. The Fermi level is set to be zero. Red and green balls represent the B and P atoms, respectively, while the Sc, C and F atoms in the Sc_2_CF_2_ monolayer are denoted by the blue, violet and orange balls, respectively.

The atomic structure and electronic properties of the BP and Sc_2_CF_2_ monolayers are depicted in Fig. 1. The BP monolayer has a planar hexagonal atomic structure with a lattice constant of 3.20 Å. The unit cell of the BP monolayer consists of one B and one P atom. The Sc_2_CF_2_ monolayer shows a layered hexagonal atomic structure with a lattice parameter of 3.26 Å, which is consistent with the previous reports.^42,43^ A layer of carbon atoms in the center is sandwiched between two layers of Sc atoms, which are functionalized by F atoms on both sides. The BP monolayer possesses a direct band gap of 0.89/1.35 eV obtained by the PBE/HSE method with both the valence-band maximum (VBM) and conduction-band minimum (CBM) at the M-point, as depicted in Fig. 1(b). This value of the band gap of the BP monolayer is in good agreement with the previous predictions.^38,50^ On the other hand, the Sc_2_CF_2_ monolayer exhibits an indirect band gap of 1.05/1.86 eV obtained by the PBE/HSE method. Such a band gap is consistent with the previous reports.^51^ The VBM of the Sc_2_CF_2_ monolayer is located at the Γ-point, while the CBM lies at the M-point, as illustrated in Fig. 1(f) and (g). Both the BP and Sc_2_CF_2_ monolayers are dynamically stable because all their phonon dispersion curves in Fig. 1(d) and (h) are positive. Therefore, both the BP and Sc_2_CF_2_ 2D materials are experimentally feasible.

**Fig. 2:**
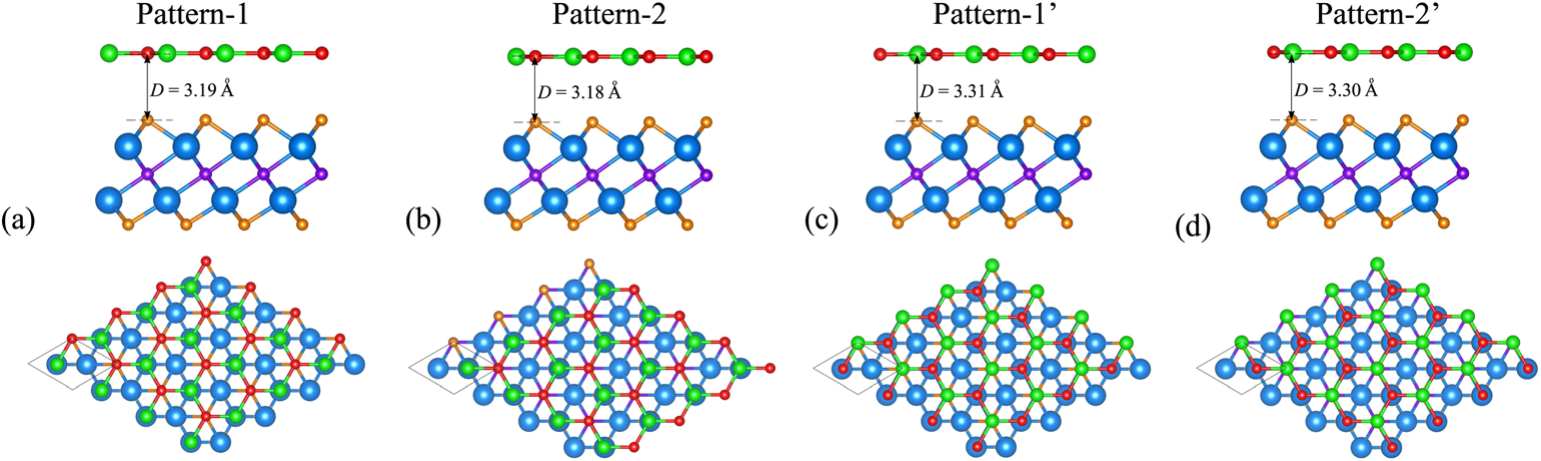
Top and side views of the atomic structures of the BP/Sc_2_CF_2_ heterostructure for different stacking patterns of (a) Pattern-1, (b) Pattern-2, (c) Pattern-1′ and (d) Pattern-2′. Red and green balls are the B and P atoms, respectively, while the Sc, C and F atoms are marked by blue, purple and orange balls, respectively.

We now build the BP/Sc_2_CF_2_ heterostructure by stacking the BP monolayer on top of the Sc_2_CF_2_ monolayer, as depicted in Fig. 2. Owing to the same lattice parameter of the BP and Sc_2_CF_2_ monolayers, the BP/Sc_2_CF_2_ heterostructure can be made by stacking a (1 × 1) unit cell of the BP layer and a (1 × 1) unit cell of the Sc_2_CF_2_ monolayer. We build four different stacking patterns of the BP/Sc_2_CF_2_ heterostructure, as shown in Fig. 2. After geometric optimization, the interlayer spacings between the BP and Sc_2_CF_2_ layers for different stacking patterns are marked in Fig. 2. Our calculations show that Pattern-2 has the lowest interlayer spacing of 3.18 Å and Pattern-1′ has the largest interlayer spacing of 3.31 Å. Furthermore, to check the structural stability of the combined heterostructure, we calculate the binding energy as follows: *E*_b_ = [*E*_BP/Sc_2_CF_2__ − (*E*_BP_ + *E*_Sc_2_CF_2__)]/*A*, where *E*_BP/Sc_2_CF_2__, *E*_BP_ and *E*_Sc_2_CF_2__ are the total energies of the BP/Sc_2_CF_2_ heterostructure, and the isolated BP and Sc_2_CF_2_ monolayers, respectively. *A* represents the surface area of the combined heterostructure. The binding energies of BP/Sc_2_CF_2_ heterostructure for the different stacking patterns, Pattern-1, Pattern-2, Pattern-1′ and Pattern-2′, are calculated to be −38.82, −39.40, −36.03 and −36.31 meV Å^−2^, respectively. One can find that Pattern-2 is the most energetically favorable stacking pattern owing to the lowest binding energy and shortest interlayer distance, as listed in Table 1. Therefore, we further focus on this most stable pattern in the following discussion.

**Table 1:** Calculated lattice parameter (*a*, Å), interlayer spacing (*D*, Å), binding energy (*E*_b_, meV Å^−2^), band gap (*E*_g_, eV) and contact type in the BP/Sc_2_CF_2_ heterostructure

	*a*	*D*	*E*_b_	*E*_g_	Contact type
Pattern-1	3.21	3.19	−38.82	0.25	Type-II
Pattern-2	3.21	3.18	−39.40	0.25	Type-II
Pattern-1′	3.21	3.31	−36.03	0.20	Type-II
Pattern-2′	3.21	3.30	−36.31	0.20	Type-II

**Fig. 3:**
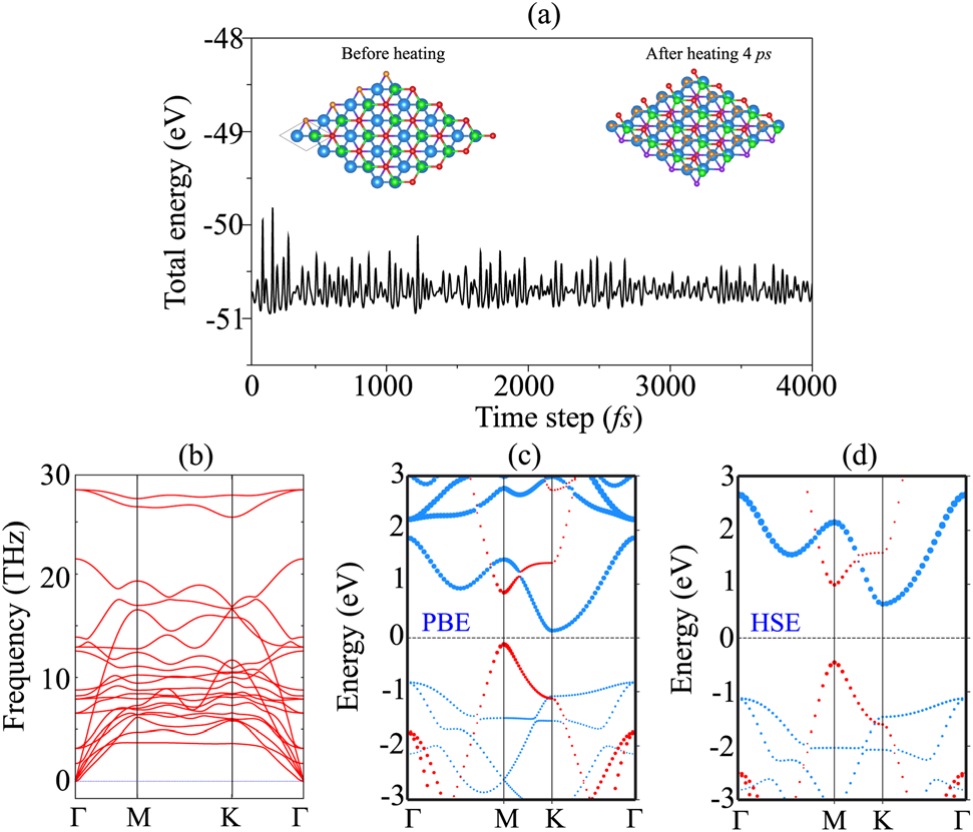
(a) The fluctuation of the total energy of the BP/Sc_2_CF_2_ heterostructure as a function of time-steps. The inset represents the atomic structure of the BP/Sc_2_CF_2_ heterostructure before and after heating for 4 ps, respectively. (b) Phonon dispersion and (c) projected PBE and (d) HSE band structures of the BP/Sc_2_CF_2_ heterostructure. The contributions of the BP and Sc_2_CF_2_ layers in the heterostructure are marked by red and blue lines, respectively.

The AIMD simulation and phonon spectra of the most stable pattern of the BP/Sc_2_CF_2_ heterostructure are displayed in Fig. 3(a) and (b) to check the thermal and dynamical stability. The AIMD simulation was performed using a 3 × 3 × 1 supercell, consisting of 72 atoms. The simulation was performed at room temperature of 300 K. One can find that there is an absence of distortions in the atomic structure of the BP/Sc_2_CF_2_ heterostructure after heating for 4 ps. In addition, the fluctuation in the total energy of the BP/Sc_2_CF_2_ heterostructure as a function of time-steps is small. All these findings confirm that the BP/Sc_2_CF_2_ heterostructure is thermally stable at room temperature. Furthermore, to check the dynamical stability of the BP/Sc_2_CF_2_ heterostructure, we calculate its phonon spectrum, as depicted in Fig. 3(b). All the phonon dispersion curves of the BP/Sc_2_CF_2_ heterostructure are positive, verifying its dynamical stability. The projected band structure of the BP/Sc_2_CF_2_ heterostructure for Pattern-2 is illustrated in Fig. 3(c). It is clear that the BP/Sc_2_CF_2_ heterostructure is a semiconductor with an indirect band gap of 0.26 eV. This band gap is smaller than those of both the constituent BP and Sc_2_CF_2_ monolayers, suggesting that the generation of the BP/Sc_2_CF_2_ heterostructure can enhance the optical absorption coefficient. In addition, we find that the combination of the BP/Sc_2_CF_2_ heterostructure gives rise to the generation of type-II (staggered gap) band alignment. The VBM of the BP/Sc_2_CF_2_ heterostructure is contributed to by the BP layer, while the CBM comes from the Sc_2_CF_2_ layer, as illustrated in Fig. 3(c). In order to obtain a more accurate band gap of the BP/Sc_2_CF_2_ heterostructure, we plot its HSE projected band structure, as depicted in Fig. 3(d). The HSE band gap of the BP/Sc_2_CF_2_ heterostructure is found to be 1.05 eV. However, the HSE method also predicts the same trend in the BP/Sc_2_CF_2_ heterostructure, confirming the reality of our considered approach. For the HSE band structure, the BP/Sc_2_CF_2_ heterostructure also generates the formation of type-II band alignment with the CBM at the K-point, and the VBM at the M-point. The photogenerated electrons and holes are spatially separated at the interface and transferred in opposite directions. Therefore, the formation of type-II band alignment in the BP/Sc_2_CF_2_ heterostructure suggests that it could be a promising candidate for photovoltaic solar cells.

**Fig. 4:**
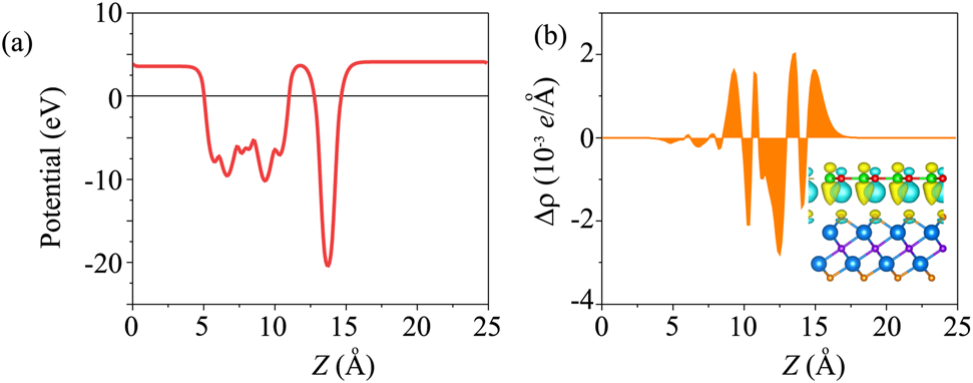
(a) Electrostatic potential and (b) planar-averaged charge-density difference of the BP/Sc_2_CF_2_ heterostructure. The inset represents the 3D visualization of the charge-density difference. The yellow and cyan regions show the charge accumulation and depletion, respectively. The isosurface is set to be 0.0025*e* Å^−3^.

The electrostatic potential of the BP/Sc_2_CF_2_ heterostructure is plotted in Fig. 4(a). The BP layer has a deeper potential than the Sc_2_CF_2_ layer. Moreover, to further understand the charge redistribution at the interface of the BP/Sc_2_CF_2_ heterostructure, we calculated the charge density difference as: Δ*ρ* = *ρ*_BP/Sc_2_CF_2__ − *ρ*_BP_ − *ρ*_Sc_2_CF_2__, where *ρ*_BP/Sc_2_CF_2__, *ρ*_BP_ and *ρ*_Sc_2_CF_2__ are the charge densities of the BP/Sc_2_CF_2_ heterostructure, and the isolated BP and Sc_2_CF_2_ monolayers, respectively. The charges are redistributed at the heterostructure interface. In addition, we find that there is an internal electric field, pointing from the Sc_2_CF_2_ layer to the BP layer.

**Fig. 5:**
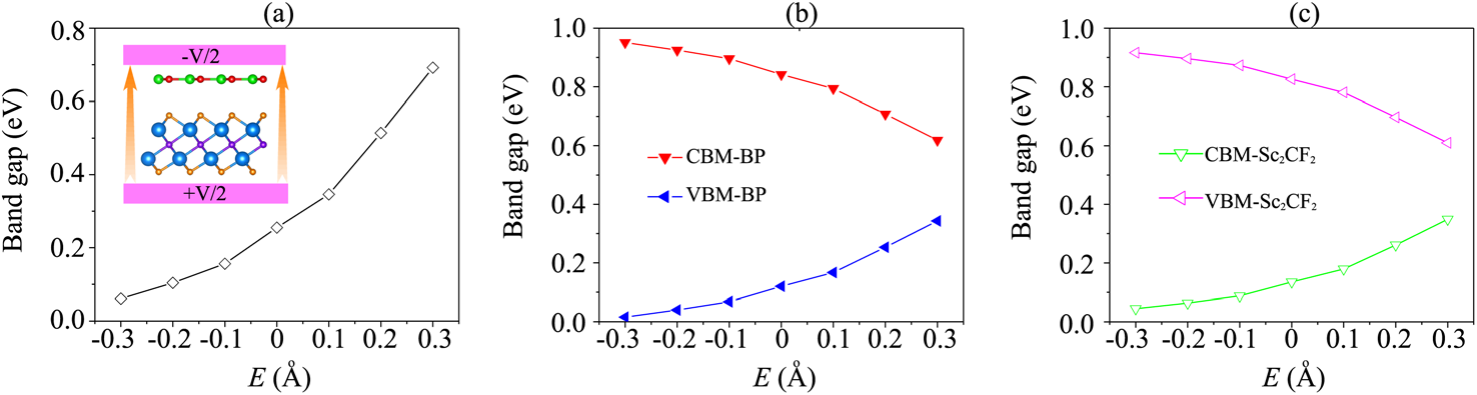
(a) The fluctuation of the band gap of the BP/Sc_2_CF_2_ heterostructure as a function of the applied electric field. The inset represents the schematic model of the applied electric field to the heterostructure. The variation of the band edges of (b) the BP layer and (c) the Sc_2_CF_2_ layer under the applied electric field.

Interestingly, the electronic properties and band alignment of heterostructures can be regulated by applying electric fields or strains.^39,52,53^ An electric field was applied along the *z*-direction of the heterostructure, as displayed in the inset of Fig. 5(a). The strength of the electric field is in the range from −0.3 V Å^−1^ to +0.3 V Å^−1^. Generally, a high-strength electric field can be generated experimentally using pulsed AC field technology.^54^ The variations of the band gap of the BP/Sc_2_CF_2_ heterostructure under an applied electric field, as well as the band edges of both BP and Sc_2_CF_2_ monolayers, are depicted in Fig. 5. It can be seen that the band gap of the BP/Sc_2_CF_2_ heterostructure is narrower under an applied negative electric field (n-*E*), whereas a positive electric field (p-*E*) leads to an enhancement in the band gap of the BP/Sc_2_CF_2_ heterostructure, as depicted in Fig. 5(a). When the applied n-*E* = −0.3 V Å^−1^, the band gap of the BP/Sc_2_CF_2_ heterostructure decreases down to 0.06 eV. One can find from Fig. 5(a) that the band gap of the BP/Sc_2_CF_2_ heterostructure can be reduced to be zero when the n-*E* is −0.4 V Å^−1^. This finding suggests that the transition from a semiconductor to a gapless semiconductor can be achieved at the BP/Sc_2_CF_2_ interface when a strong n-*E* of −0.4 V Å^−1^ is applied. On the other hand, the band gap of the BP/Sc_2_CF_2_ heterostructure reaches 0.69 eV when the applied p-*E* = +0.3 V Å^−1^.

**Fig. 6:**
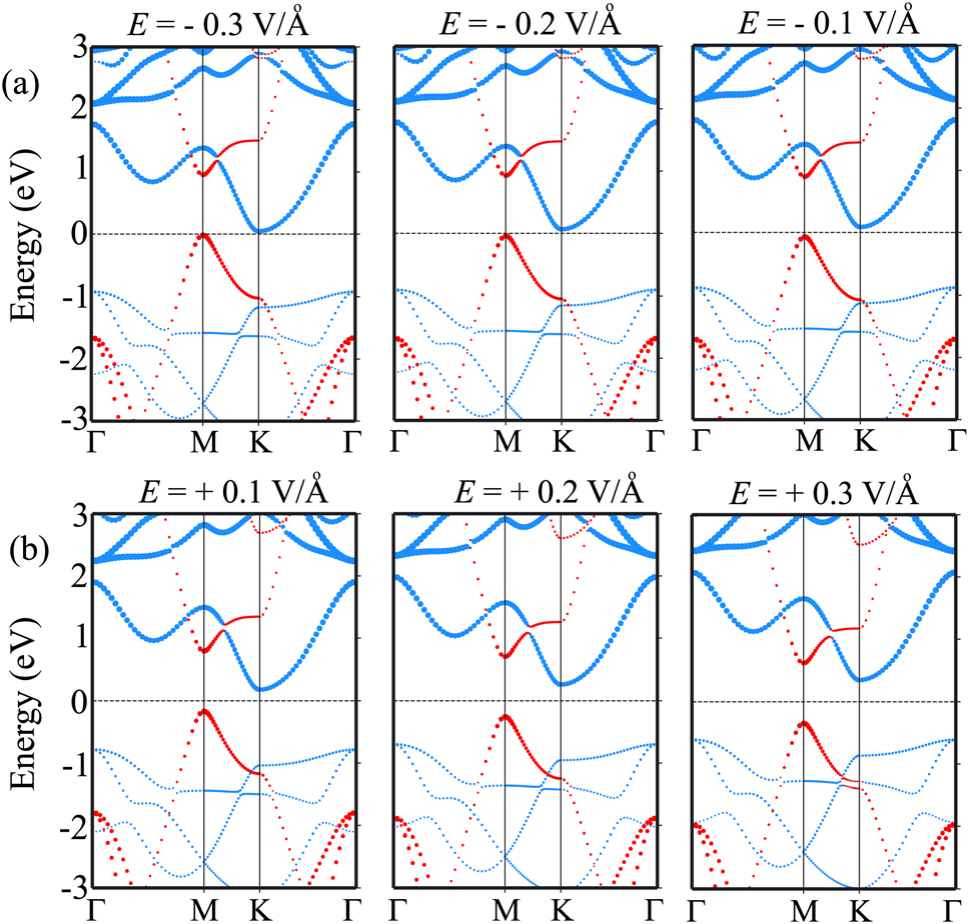
The projected band structures of the BP/Sc_2_CF_2_ heterostructure under (a) negative and (b) positive electric field. The contributions of the BP and Sc_2_CF_2_ layers are marked by red and blue lines, respectively.

Furthermore, it is obvious that the changes in the band gap of the BP/Sc_2_CF_2_ heterostructure under applied *E* are totally related to the variations of the band edges of the constituent BP and Sc_2_CF_2_ monolayers. We therefore plot the variations of the band edges of BP and Sc_2_CF_2_ monolayers, as well as the projected band structures of the BP/Sc_2_CF_2_ heterostructure, as a function of applied *E*, as illustrated in Fig. 5(b and c) and Fig. 6, respectively. As discussed above, the BP/Sc_2_CF_2_ heterostructure forms type-II band alignment, in which the BP layer contributes to the VBM, while the Sc_2_CF_2_ layer contributes to the CBM. With an applied n-*E*, both the VBM energy of the BP layer and CBM energy of the Sc_2_CF_2_ layer are narrowed, giving rise to the reduction in the band gap of the BP/Sc_2_CF_2_ heterostructure. From the projected band structures of the BP/Sc_2_CF_2_ heterostructure with an applied n-*E* in Fig. 6(a), we find that the CBM dominated by the Sc_2_CF_2_ layer at the K-point and the VBM dominated by the BP layer at the M-point are moved towards the Fermi level. The band gap of the BP/Sc_2_CF_2_ heterostructure is thus narrowed under an applied n-*E*. When the n-*E* is smaller than −0.3 V Å^−1^, both the VBM and CBM shift towards the Fermi level and cross the Fermi level, resulting in the transition from a semiconductor to a gapless semiconductor. On the other hand, when a p-*E* is applied, the energy of the VBM originating from the BP layer and the energy of the CBM originating from the Sc_2_CF_2_ layer in the BP/Sc_2_CF_2_ heterostructure shift far from the Fermi level. Therefore, the band gap of the BP/Sc_2_CF_2_ heterostructure is increased. The projected band structures of the BP/Sc_2_CF_2_ heterostructure are displayed in Fig. 6(b). We observe that both the VBM and CBM of the BP/Sc_2_CF_2_ heterostructure move far from the Fermi level, giving rise to an enhancement in its band gap. More interestingly, our calculations demonstrate that the application of the higher p-*E* can result in the transformation from type-II to type-I band alignment in the BP/Sc_2_CF_2_ heterostructure. The nature of the changes in the band gap of the BP/Sc_2_CF_2_ heterostructure is due to the Stark effect. In addition, one can find that the direction of the external and internal electric field is the same, resulting in an enhancement of strength. Thus, the band gap of the BP/Sc_2_CF_2_ heterostructure is reduced when a negative electric field is applied. On the other hand, the direction of the positive and internal electric field is opposite, leading to an enhancement in the band gap of the BP/Sc_2_CF_2_ heterostructure. However, the large strength of the electric field would be quite difficult to generate experimentally. Therefore, we can conclude that the application of electric fields not only leads to the change in the band gap, but also gives rise to the semiconductor–gapless semiconductor transition and type-II to type-I transformation in the BP/Sc_2_CF_2_ heterostructure.

**Fig. 7:**
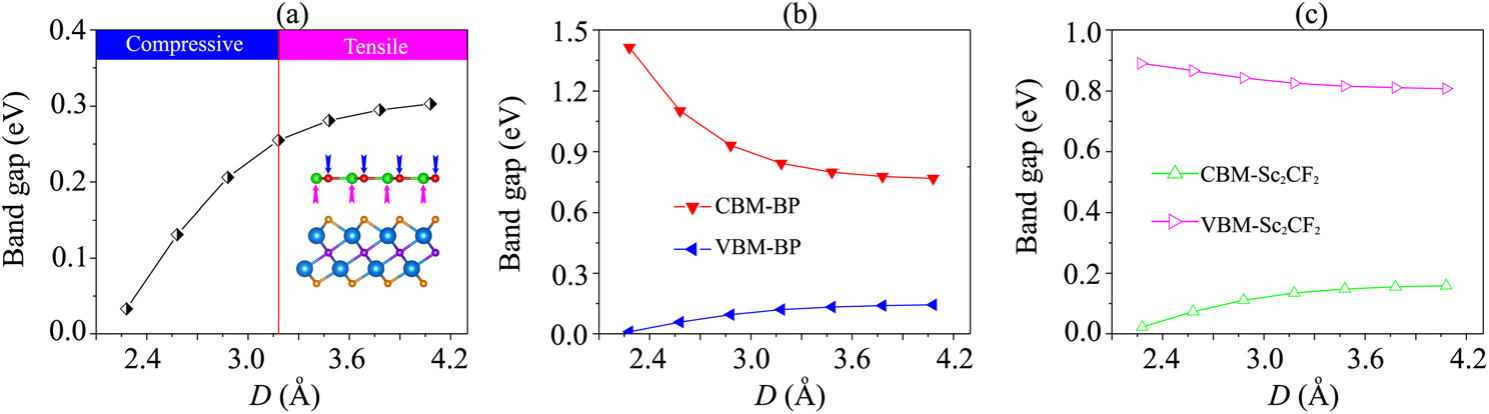
(a) The fluctuation of the band gap of the BP/Sc_2_CF_2_ heterostructure as a function of interlayer spacing. The inset represents the schematic model of applied strain by changing the interlayer spacing. The variation of the band edges of (b) the BP layer and (c) the Sc_2_CF_2_ layer as a function of interlayer spacing.

**Fig. 8:**
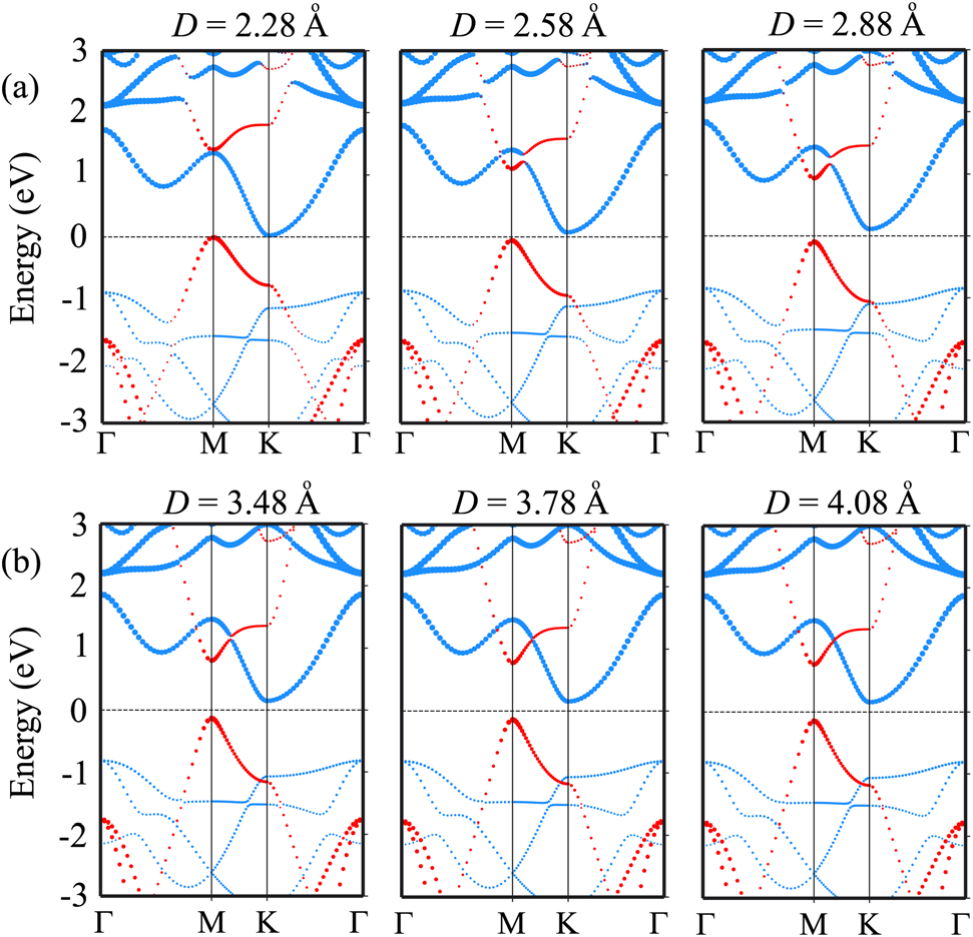
The projected band structures of the BP/Sc_2_CF_2_ heterostructure under (a) compressive strain by reducing the interlayer spacing, and (b) tensile strain by increasing the interlayer spacing in the BP/Sc_2_CF_2_ heterostructure. The contributions of the BP and Sc_2_CF_2_ layers are marked by red and blue lines, respectively.

We further investigate the effects of the interlayer coupling on the electronic properties and contact types of the BP/Sc_2_CF_2_ heterostructure. The schematic model of applied strain by changing the interlayer spacing *D* is depicted in the inset of Fig. 7(a). The stronger interlayer coupling corresponds to compressive strain, while the weaker interlayer coupling corresponds to tensile strain. One can find that the stronger the interlayer coupling is, the smaller the band gap will be, as depicted in Fig. 7(a), whereas the weaker the interlayer coupling is, the larger the band gap will be. This finding demonstrates that compressive strain leads to a reduction in the band gap, while tensile strain gives rise to an enhancement in the band gap of the BP/Sc_2_CF_2_ heterostructure. The physical nature of the change in the band gap can be described by analyzing the shift of the band edges of the BP/Sc_2_CF_2_ heterostructure relative to the Fermi level, as illustrated in Fig. 7(b and c) and Fig. 8. It is obvious that the fluctuation of the VBM originating from the BP layer and the CBM originating from the Sc_2_CF_2_ layer is the same. The compressive strain reduces the energy of the VBM and CBM band edges, giving rise to the narrower band gap. On the other hand, the tensile strain enhances the energy of the VBM and CBM band edges, leading to the greater band gap of the BP/Sc_2_CF_2_ heterostructure. Furthermore, when the interlayer coupling is stronger, both the VBM and CBM of the BP/Sc_2_CF_2_ heterostructure shift towards the Fermi level, whereas they move in the opposite direction when the interlayer coupling is weakened. Therefore, we can conclude that the interlayer coupling can be used effectively for tuning the electronic properties of the BP/Sc_2_CF_2_ heterostructure.

## 4 Conclusions

In conclusion, we have constructed a BP/Sc_2_CF_2_ heterostructure and explored its electronic properties and band alignment, as well as the effects of applying an electric field and modifying the interlayer coupling, using first-principles prediction. BP/Sc_2_CF_2_ heterostructures are predicted to be energetically, thermally and dynamically stable. All the stacking patterns of the BP/Sc_2_CF_2_ heterostructure exhibit a semiconducting nature with direct band gaps and formation of type-II band alignment. The type-II BP/Sc_2_CF_2_ heterostructure is useful for the design of photovoltaic solar cells because of the spatial separation of the photogenerated electrons and holes. Furthermore, the electronic characteristics and band alignments of the BP/Sc_2_CF_2_ heterostructure are adjustable by applying an electric field and modifying the interlayer coupling. The transition from a semiconductor to a gapless semiconductor and from type-II to type-I band alignment can be achieved under an applied electric field, while tunable electronic properties of the BP/Sc_2_CF_2_ heterostructure are observed by adjusting the interlayer coupling in the BP/Sc_2_CF_2_ heterostructure. Our findings suggest that the BP/Sc_2_CF_2_ heterostructure with type-II band alignment could be a promising candidate for photovoltaic solar cells.

## Data availability

The data that support the findings of this study are available from the corresponding author upon reasonable request.

## Conflicts of interest

There are no conflicts to declare.

## Supplementary Material
